# The Regulatory Circuit Underlying Downregulation of a Type III Secretion System in *Yersinia enterocolitica* by Transcription Factor OmpR

**DOI:** 10.3390/ijms23094758

**Published:** 2022-04-26

**Authors:** Marta Nieckarz, Karolina Jaworska, Adrianna Raczkowska, Katarzyna Brzostek

**Affiliations:** Department of Molecular Microbiology, Institute of Microbiology, Faculty of Biology, University of Warsaw, Ilji Miecznikowa 1, 02-096 Warsaw, Poland; nieckarzmarta@gmail.com (M.N.); k.jaworska3@uw.edu.pl (K.J.)

**Keywords:** *Yersinia enterocolitica*, OmpR regulator, T3SS

## Abstract

In a previous study, differential proteomic analysis was used to identify membrane proteins of the human enteropathogen *Yersinia enterocolitica*, whose levels are influenced by OmpR, the transcriptional regulator in the two-component EnvZ/OmpR system. Interestingly, this analysis demonstrated that at 37 °C, OmpR negatively affects the level of over a dozen Ysc-Yop proteins, which constitute a type III secretion system (T3SS) that is essential for the pathogenicity of *Y. enterocolitica*. Here, we focused our analysis on the role of OmpR in the expression and secretion of Yops (translocators and effectors). Western blotting with anti-Yops antiserum and specific anti-YopD, -YopE and -YopH antibodies, confirmed that the production of Yops is down-regulated by OmpR with the greatest negative effect on YopD. The RT-qPCR analysis demonstrated that, while OmpR had a negligible effect on the activity of regulatory genes *virF* and *yscM1*, it highly repressed the expression of *yopD*. OmpR was found to bind to the promoter of the *lcrGVsycD-yopBD* operon, suggesting a direct regulatory effect. In addition, we demonstrated that the negative regulatory influence of OmpR on the Ysc-Yop T3SS correlated with its positive role in the expression of *flhDC*, the master regulator of the flagellar-associated T3SS.

## 1. Introduction

*Yersinia enterocolitica* is a human enteropathogen causing a broad range of gastrointestinal diseases [1,2]. This species belongs to the genus *Yersinia*, which includes two other pathogenic species: the plague bacillus *Y. pestis* and *Y. pseudotuberculosis*, another enteropathogen [3]. *Y. enterocolitica* synthesizes numerous chromosomally- and plasmid pYV-encoded virulence factors. The chromosomally-encoded proteins include adhesins Ail, Inv and Myf, phospholipase A and heat-stable enterotoxin (Yst), which allow the bacterial cells to adhere to and invade the intestinal epithelium and/or colonize the peripheral tissues [1,4]. Plasmid pYV encodes adhesin YadA and the Ysc-Yop Type III Secretion System (T3SS) [5]. This T3SS functions in all three pathogenic *Yersinia* species and is essential for the ability to cause disease [6,7]. The expression of these virulence determinants is altered in response to changes in the physicochemical parameters of the environment [8]. The Ysc-Yop T3SS of *Yersinia* is a well-characterized archetype for this type of secretion and cell contact-dependent translocation process, which is also found in other pathogenic bacteria, including *Salmonella* and *Shigella* [9,10,11]. The *Yersinia* T3SS is comprised of the complex Ysc protein secretion apparatus (often called the injectosome), which resembles a syringe with a base structure that spans the inner and outer membranes, and a needle-like structure with a hollow channel that protrudes outside the bacterial cell [10]. Upon contact with a target host cell, the translocator proteins YopB/YopD, with the assistance of LcrV located at the tip of the T3SS needle, create a pore in the host cell membrane which enables the injection of effectors: a number of anti-host proteins known as Yops (*Yersinia* outer proteins) [5,7,12]. The main function of *Yersinia* Yop effector proteins is to inhibit the host organism’s defense reaction [13]. Yops disrupt eukaryotic signal transduction mechanisms, leading to disorganization of the host cell cytoskeleton (YopH, YopE, YopT, YopO/YpkA) and inhibition of the inflammatory and immune responses (YopP/J and YopH). The secretion of Yop proteins requires the presence of small cytoplasmic chaperones, called Syc proteins [14]. The component T3SS proteins are the most important virulence factors of *Y. enterocolitica* and are conserved among pathogenic *Yersinia* species [5,7,13]. Various models have been proposed for the secretion/translocation mechanism. In the prevailing model of T3SS function, secretion and translocation of effector Yops occur in a “one-step” process. The type III secretion apparatus, consisting of approximately 20 proteins, mediates substrate protein selection, which involves docking of substrate proteins, often with their cognate secretion chaperones, to the export apparatus and their subsequent unfolding. This enables secretion and direct translocation from the bacterial cell to the host cytosol [10]. In addition, the T3SS can discriminate between different substrate classes [15]. Once the assembly of the injectosome basal-body complex has been completed, the T3SS switches substrate specificity from early-type substrates (components of the secretion apparatus necessary for needle assembly) to the middle-type substrates (translocators YopB/YopD that together with the needle cap protein LcrV create a pore in the host cell membrane), and finally to the late-type substrates (Yop proteins). This mechanism prevents premature secretion of the Yop effector proteins before the injectosome has been fully assembled. More recently, a “two-step” model of T3SS-dependent protein translocation was proposed [16,17]. According to this model, the middle and late Yops substrates, secreted by the T3SS, associate with the bacterial outer membrane. In the next step, i.e., when the bacterium binds tightly to the host cell, the surface-localized Yops are released and efficient translocation of the effectors across the plasma membrane occurs via a pore created by the translocators.

The regulation of Ysc-Yop T3SS expression in pathogenic *Yersinia* is highly complex and tightly connected with the secretion process, which is triggered at a temperature of 37 °C in a calcium-deficient medium in vitro or by close contact between the bacterial and eukaryotic cells in vivo [18,19,20,21,22]. Several chromosomally- and pYV-encoded regulators have been identified that play a role in regulating Ysc-Yop expression/secretion [23]. Temperature-controlled upregulation of the T3SS is mediated by the chromosomally-encoded nucleoid-associated YmoA protein, a thermosensitive repressor of the *virF* gene located on plasmid pYV, which encodes transcriptional activator VirF (LcrF in *Y. pseudotuberculosis*) [24,25,26]. VirF exhibits sequence similarity to the AraC activator of *Escherichia coli* and has been shown to interact with several promoters of T3SS-related genes [25,26,27,28,29]. The regulator of the so-called “low calcium response” phenotype has been identified as LcrQ in *Y. pseudotuberculosis* (YscM1 in *Y. enterocolitica*) [18,30]. The protein, LcrQ, has been proposed as a negative regulator whose secretion by T3SS relieves repression of Yops, which permits further upregulation of their synthesis and secretion [21,31,32]. It was recently shown that negative regulation of LcrQ is strictly dependent on the YopD-LcrH complex that is involved in repressing *lcrF* expression at the post-transcriptional level [33,34]. Several other regulatory elements of T3SS are encoded on pYV, including the YopN-TyeA complex and Yops chaperones [15,22]. The molecular mechanisms integrating other environmental signals involve several plasmid-independent regulatory elements. These include the histone-like DNA-binding protein YmoA, which is a repressor of *lcrF*; the CpxRA two-component regulatory system (TCS) involved in negative regulation of the Ysc-Yop T3SS [35,36]; and an Rcs phosphorelay system that positively regulates this T3SS by activating *lcrF* expression [37].

The EnvZ-OmpR constitutes an important regulatory pathway in enterobacterial cells that mediates a variety of adaptive responses to changes in environmental signals [38,39,40]. This TCS consists of the sensor protein EnvZ, with kinase and phosphatase activity, that monitors changes in the environment (e.g., in osmolarity or pH) and controls the cellular level of phosphorylated OmpR, which acts as the response regulator by modulating the transcription of target genes [41,42,43]. An EnvZ/OmpR system has been identified in *E. coli* and in several pathogenic enterobacteria, including *Shigella* and *Salmonella*, where it plays an important role in controlling various cellular functions, including virulence [44,45,46,47,48,49,50,51]. In the last few years, a number of OmpR-regulated genes have been revealed using the DNA microarray and ChIP-chip approach, which confirms that the binding of OmpR to target genes can control the level of proteins involved in many physiological functions in bacterial cells [52,53]. The EnvZ/OmpR TCS has also been identified in pathogenic species of the genus *Yersinia* [54,55,56,57]. The physiological consequences of the loss of the OmpR protein studied in *Y. enterocolitica* have revealed the role of this regulator in the adaptation of this bacterium to multiple environmental stresses [58,59]. OmpR was also identified as the regulator of biofilm formation and the adherent-invasive abilities of this bacterium [60]. Moreover, it has been shown that OmpR plays a role in coordinating the motility of *Y. enterocolitica* by positively regulating transcription of the operon encoding FlhDC, the master activator of the flagellar regulon [61]. In addition, a correlation between serum resistance of *Y. enterocolitica* and OmpR-dependent changes in the outer membrane proteins YadA, Ail and OmpX, as well as in lipopolysaccharides, was indicated [62]. More recent studies have demonstrated the impact of OmpR on iron homeostasis by controlling levels of the Fur repressor and the outer membrane receptors of heme/iron-acquisition systems [63,64].

The present study examined the influence of the OmpR regulator on the expression of Ysc-Yop T3SS proteins. Previously, we applied shotgun label-free mass spectrometry to quantify the differences in protein abundance within the outer membrane (OM) proteome of *Y. enterocolitica* in response to the presence of OmpR in cells grown under different temperatures, osmolarity and pH conditions [65]. This analysis produced a dataset of over one hundred membrane proteins or proteins functionally connected with the OM fraction that are involved in various biological processes, and whose abundance is affected by OmpR. This proteomic study revealed OmpR-dependent changes in several proteins belonging to the “Pathogenesis” Gene Ontology (GO) category, including components of the Ysc-Yop T3SS. Here, we characterize the influence of OmpR on the expression of T3SS proteins.

## 2. Results

### 2.1. Proteomic Analysis Reveals the Impact of OmpR on the Abundance of Y. enterocolitica Pathogenicity Factors

**Figure 1:**
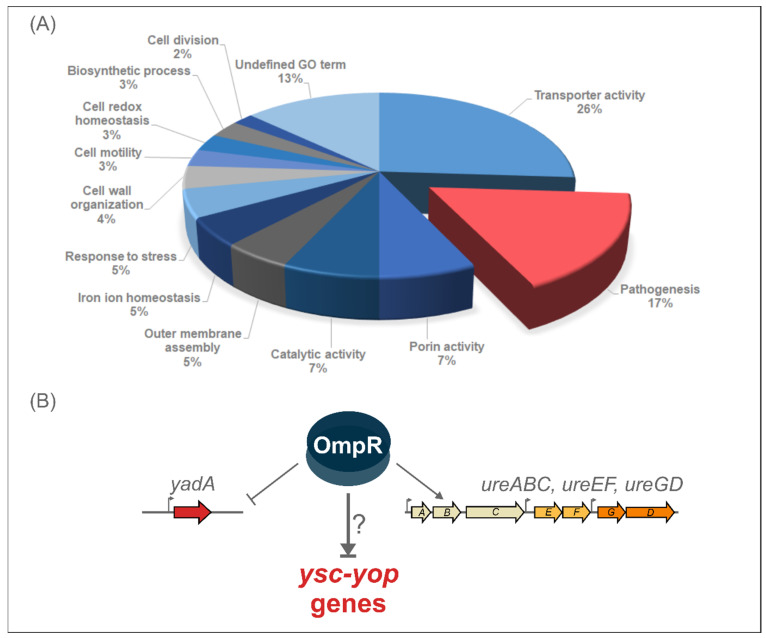
(**A**) Classification of proteins differentially expressed in wild-type *Y*. *enterocolitica* strain Ye9 compared with OmpR-deficient mutant AR4 under different growth conditions (temperature, osmolarity and pH) according to Gene Ontology (GO) biological process. The classification of the OmpR-regulated proteins is based on information from the BioCyc database collections, the Uniprot databases and the literature [65]. (**B**) Scheme for OmpR-dependent regulation of pathogenesis genes in *Y*. *enterocolitica* 2/O:9 at 37 °C. OmpR downregulates the major adhesin gene *yadA* [65], but positively affects the expression of *ureABC*, *ureEF* and *ureGD* in *Y*. *enterocolitica* strain Ye9 [66]. The influence of OmpR on the Ysc-Yop T3SS genes is investigated in this work.

Label-free quantitative mass spectrometry of in-solution trypsin digestion products (shotgun procedure) was applied for the proteomic analysis of the outer membrane-enriched sarkosyl-insoluble fractions (OMsl fraction) of *Y. enterocolitica* wild-type strain Ye9 (bioserotype 2/O:9) and the *ompR* deletion mutant of this strain (AR4), cultured in LB medium (standard conditions); LB supplemented with NaCl (386 mM), or in LB at pH 5.5, at both 26 °C and 37 °C [65]. The *ompR* mutant (Δ*ompR*::Km) AR4 was constructed by a reverse genetics PCR-based strategy [59]. The procedure used for the isolation of OMsl was designed to obtain the highest number of proteins in a highly reproducible manner from multiple biological samples handled in parallel. This analysis revealed over one hundred membrane proteins or proteins functionally connected with the OM fraction that are involved in various biological processes and are affected by OmpR. Proteins belonging to the “Pathogenesis” Gene Ontology (GO) category accounted for 17% of the OmpR-regulated targets (Figure 1). The list of proteins involved in pathogenesis and differentially regulated by OmpR under the applied environmental conditions, was dominated by pYV-encoded components of the Ysc-Yop T3SS. These proteins were more abundant in the outer membrane-enriched fractions of the *ompR* mutant than in those of wild-type Ye9N cells grown at 37 °C under all tested environmental conditions. Another protein that was more abundant at 37 °C in the *ompR* mutant was the pYV-encoded adhesin YadA, and we have characterized the influence of OmpR on *yadA* gene expression at the transcriptional, post-transcriptional and protein levels [65]. Moreover, on the list of OmpR-regulated proteins were structural components of urease (UreA and UreC, and accessory protein UreG), an important enzyme for enteric pathogens, including *Yersinia*, that promotes persistence in environmental niches of low pH. Our recent study showed that the urease genes in *Y. enterocolitica* Ye9 are organized in three *ure* transcriptional units (*ureABC*, *ureEF* and *ureGD*), and that OmpR has a positive impact on their expression at 37 °C [66]. Urease genes are also directly and positively regulated by OmpR in *Y. pseudotuberculosis* [54].

### 2.2. Impact of OmpR on the T3SS Proteome under Different Environmental Conditions

**Figure 2:**
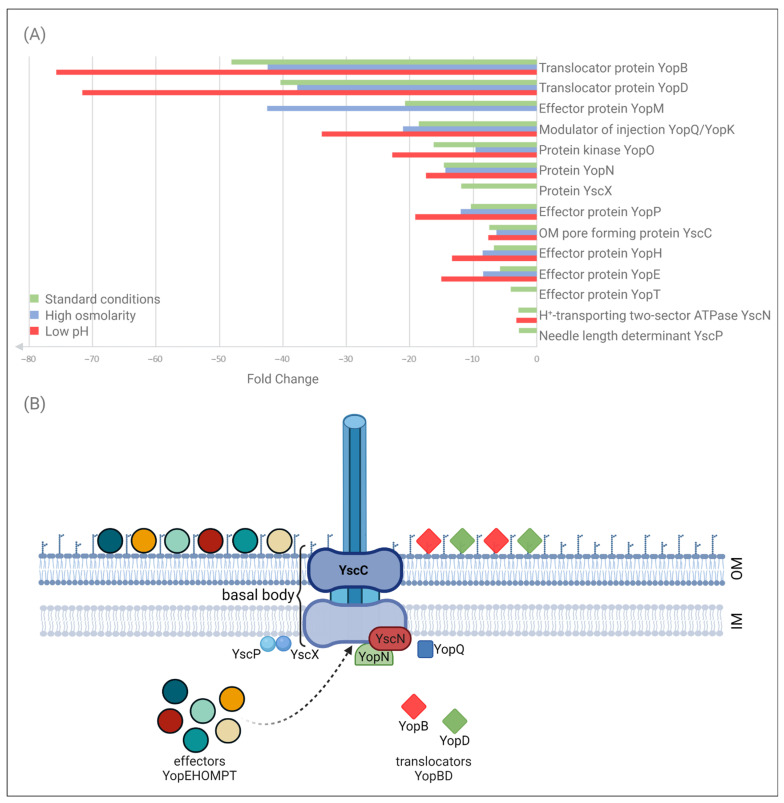
Proteins of the *Y. enterocolitica* Ysc-Yop T3SS whose abundance differs significantly between wild-type strain Ye9 and *ompR* mutant AR4. (**A**) The chart shows the AR4:Ye9 protein abundance ratios under different growth conditions. Green indicates standard conditions (LB medium); blue indicates high osmolarity (LB supplemented with NaCl to 386 mM) and red indicates low pH (LB adjusted to pH 5.0), with incubation at 37 °C. (**B**) Diagram of the *Y. enterocolitica* cell wall showing the Ysc secretory apparatus and its substrates before target cell contact, according to the two-step model of secretion. The proteins displaying increased abundance in the *ompR* mutant compared with the wild-type strain when grown in LB medium at 37 °C are marked. Protein YscC forms a ring in the bacterial outer membrane (the OM ring) and creates the basal body of the secretory apparatus. YscN, located on the cytosolic face of the basal body, is the ATPase driving the secretion of substrates. YscP and YscX are categorized as early secretion substrates: YscP participates in needle assembly and as a substrate specificity switch, YscX is required for the export of early substrates. YopN acts to block Yops export. YopQ may interact with YopD to influence the function of the injectosome. YopB and YopD are translocators. YopEHOMPT is the effector Yops. Created with BioRender.com (accessed on 24 February 2022).

We focused our analysis on the components of Ysc-Yop T3SS that showed significant differences in abundance in the *ompR* mutant AR4 compared with the wild-type Ye9, cultured at 37 °C under different osmolarity and pH conditions (Table 1, Figure 2). The T3SS system includes approximately 20 proteins that play a central role in the interaction between *Yersinia* and their eukaryotic hosts by allowing the bacteria to inject Yop effector proteins into the cytosol of target cells [10]. Differential analysis (qualitative and quantitative) led to the identification of 14 T3SS proteins under the regulatory influence of OmpR. The effect of OmpR on the T3SS proteome was observed at 37 °C but not at 26 °C, except in the case of secretin YscC (see below). The strongest upregulation in the *ompR* mutant AR4 at 37 °C was observed for the proteins YopB and YopD involved in the translocation of effector Yops into host cells. The respective magnitude of the upregulation of these proteins varied from ~48 and 40-fold in standard LB up to ~75 and 72-fold in LB at low pH. Six effector proteins were upregulated in the *ompR* mutant at 37 °C: YopM, YopO, YopP, YopH, YopE and YopT (ranked from highest to lowest fold change in LB). Their abundance varied from ~4–42-fold according to the osmolarity and pH conditions. For certain proteins, the magnitude of this upregulation doubled under conditions of high osmolarity (YopM) or low pH (YopE, YopH and YopT). In addition to the Yop translocators and effectors, some components of the Ysc secretory machinery were identified as being regulated by OmpR. As mentioned above, secretin YscC, an integral outer membrane protein forming the basal body of T3SS, the major component of type III secretion systems [67,68], was upregulated in the *ompR* mutant at both temperatures. The magnitude of this upregulation varied from ~6–16-fold (at 26 °C) according to the osmolarity and pH conditions [66; data not shown]. At 37 °C, the increase was ~7-fold, independent of the osmolarity or pH (Table 1). The abundance of five other differentially expressed proteins was increased in the *ompR* mutant background in at least one of the tested growth media: YscN, an ATPase located on the cytosolic face of the basal body, necessary for the secretion of substrates [69] (~2-fold increase); YscP, a participant in needle assembly [70] (~2-fold); YscX, required for the export of substrates [68] (~11-fold); YopN, causing blockage of Yops export [71] (~2-fold increase); and YopQ, which may interact with YopD to influence the function of the injectosome [15]. The YopQ protein (YopK in *Y. pseudotuberculosis*) was more abundant in the *ompR* mutant compared with the wild-type strain, and upregulation varied from ~18-fold under standard conditions to 21-fold and ~34-fold under high osmolarity and low pH conditions, respectively.

Together, these proteomic data showed that in cells grown at 37 °C, OmpR negatively influences the abundance of Ysc-Yop T3SS proteins to different extents depending on the osmolarity and pH conditions.

### 2.3. Evaluation of the Abundance of Yops in the OmpR-Deficient Strain

**Figure 3:**
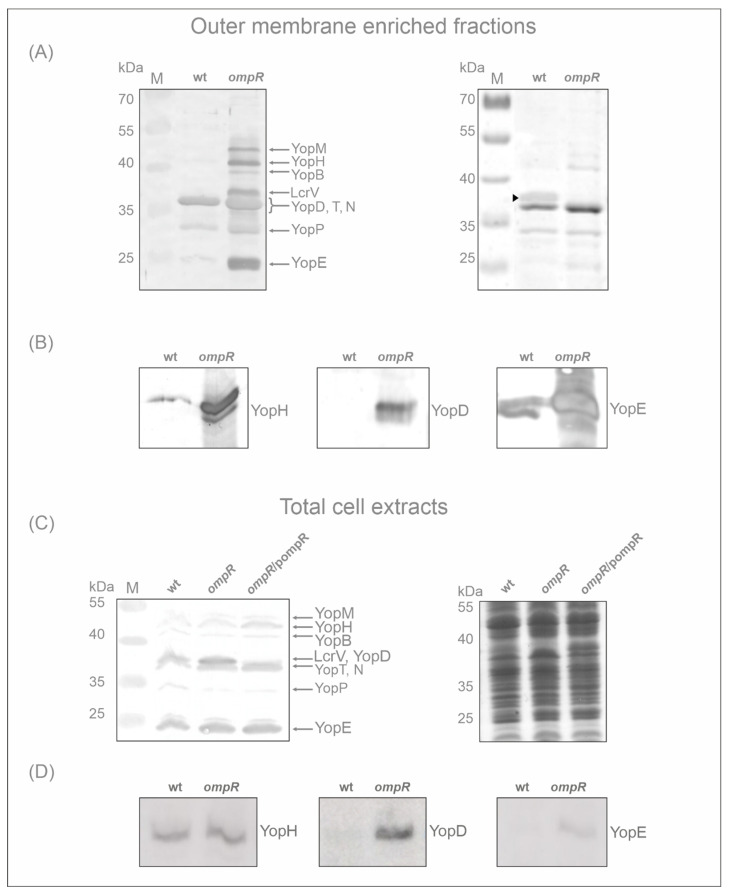
Effect of OmpR on the expression of Yop proteins. Yop protein abundance in *Y. enterocolitica* Ye9 (wt) and AR4 (*ompR*) strains were analysed by 12% SDS-PAGE and Western blotting. (**A**) Immunodetection of Yop proteins in the outer membrane-enriched sarkosyl resistant fractions utilized for MS proteomic analysis (OMsl fraction). The left panel shows a Western blot analysis of Yop proteins with an anti-Yops antiserum. The Coomassie blue-stained SDS-PAGE gel is shown in the right panel. Strains were grown in standard LB medium at 37 °C. The position of the OmpC/F porin band is marked by an arrowhead on the stained gel image. The positions of the Yops (YopM, -H, -B, -D, -T, -N, -P and -E) and LcrV are indicated. M, molecular weight standards (PageRuler Prestained Protein Ladder, Thermo Fisher Scientific). (**B**) Immunodetection of YopH, YopD and YopE proteins in the OMsl fraction of the wild-type Ye9 and the *ompR* mutant AR4 grown in standard LB medium at 37 °C, performed with specific anti-YopH, -YopD and -YopE polyclonal antibodies. (**C**) Immunodetection of Yop proteins in total cell extracts with anti-Yops antiserum. The strains (wt, *ompR* and *ompR*/pompR) were grown in standard LB medium at 37 °C and equivalent whole-cell lysate samples were analysed. The left panel shows a Western blot analysis of Yop proteins. The Coomassie blue-stained SDS-PAGE gel is shown in the right panel. The positions of the Yops (YopM, -H, -B, -D, -T, -N, -P, -E) are indicated. M, molecular weight standards (PageRuler Prestained Protein Ladder, Thermo Fisher Scientific). (**D**) Immunodetection of the YopH, YopD and YopE proteins in total cell extracts of the wild-type strain and the *ompR* mutant grown in standard LB medium at 37 °C, using specific anti-YopH, -YopD and -YopE antibodies. Equivalent whole-cell lysate samples were analysed.

Proteomic analysis of the OMsl fraction showed substantial alterations in the abundance of Yop proteins (translocators and effectors) resulting from the loss of regulator OmpR. To confirm this observation, we used an antiserum directed against Yop proteins to perform a Western blot analysis of the OMsl samples used for the MS studies (Figure 3A). Strong bands of highly abundant Yops were detected in the sample prepared from the *ompR* mutant AR4 grown in LB at 37 °C. The band intensities were much greater than in the equivalent wild-type Ye9 sample. As a loading control, the Coomassie blue-stained gel of OMsl samples is shown in the right-hand panel of Figure 3A. As expected, an OmpC/OmpF porin band, located at approx. 35 kDa on the stained gel (the porins are not resolved into separate bands under these conditions), is present in the wild-type strain but absent from the *ompR* mutant sample. OmpR is an activator of *ompC/ompF* expression in *E. coli* and *Y. enterocolitica*, thus this result confirms the phenotype of the *ompR* deletion mutant [72]. Next, specific antibodies were used for the immunodetection of two effector Yops, i.e., YopH and YopE, and translocator YopD in the OMsl samples. This analysis confirmed the higher levels of these Yop proteins in the *ompR* mutant background compared with the wild-type when both strains were grown in LB at 37 °C (Figure 3B). 

To further analyse the role of OmpR in the regulation of the Ysc-Yop T3SS, we examined the abundance of Yop proteins in total cell extracts of strains differing in OmpR content, grown in LB at 37 °C: wild-type Ye9, *ompR* mutant AR4, and AR4 carrying the *ompR* allele in trans on plasmid pompR [65]. Surprisingly, Western blot analyses of these samples with anti-Yops antiserum did not show the striking differences in the level of Yops between the wild-type strain and the *ompR* mutant seen in OMsl samples (Figure 3C). Interestingly, only the band at the position corresponding to YopD and/or LcrV was clearly stronger in the total cell extracts of the *ompR* strain. Complementation of the *ompR* deletion mutant AR4 with pompR restored the level of the Yops to that observed in wild-type strain Ye9. Since YopD, together with the other translocatory protein YopB, showed the highest abundance of all Ysc-Yop T3SS proteins in OMsl samples of the *ompR* strain according to the proteomic data, i.e., a 40-fold increase for YopD in LB medium (Table 1), the strong immunoreactive band in the *ompR* mutant cell extract was assumed to be YopD. This was confirmed by the result of the Western blot with a polyclonal anti-YopD antibody. In comparison, the bands reacting with antibodies specific to YopH and YopE were of similar intensities in the wild-type and mutant cell extracts (YopH) or only slightly increased by the absence of OmpR (YopE) (Figure 3D). 

**Figure 4:**
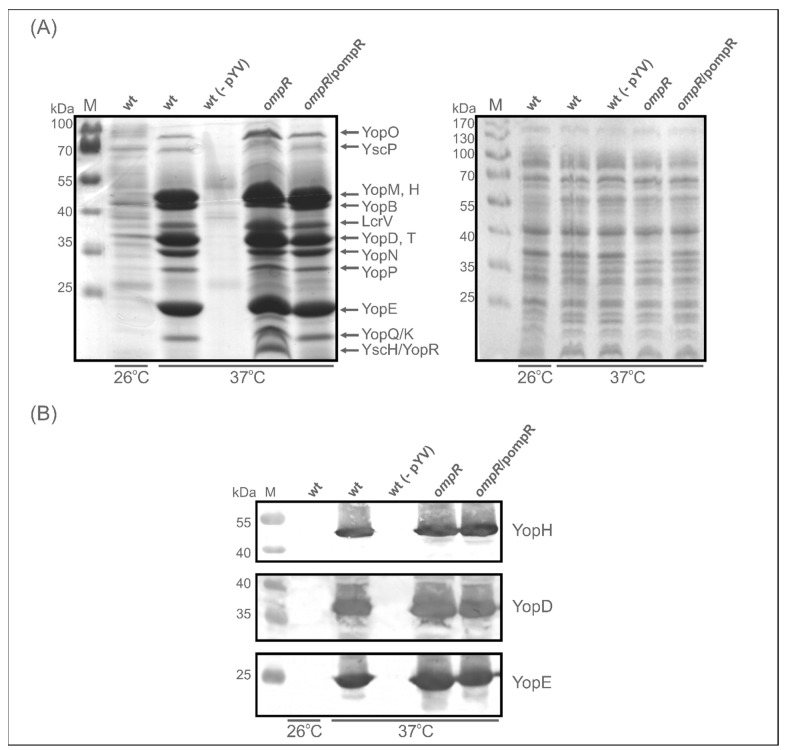
Effect of OmpR on the secretion of Yop proteins examined using 12% SDS-PAGE and Western blotting. (**A**) Yop protein abundance in the culture supernatants of *Y. enterocolitica* strains Ye9 (wt), Ye9 devoid of pYV: wt (-pYV), AR4 (*ompR*) and complemented strain AR4 (*ompR*/pompR), grown under Yop-inducing conditions, i.e., in BHI medium in the absence of Ca^2+^ (BHI-MOX) at 37 °C or at 26 °C (only Ye9 (wt)). Proteins from culture volumes containing equal numbers of cells were analysed. Coomassie blue-stained gels of supernatants (left panel) or whole-cell extracts (right panel) of *Y. enterocolitica* strains are shown. The positions of the secreted proteins (YopO, YscP, YopM, -H, -B, LcrV, YopD, -T, -N, -P, -E, -K/Q, YscH/YopR) are indicated. M, molecular weight standards (PageRuler Prestained Protein Ladder, Thermo Fisher Scientific). (**B**) Western blot analysis of YopH, YopD and YopE proteins in the supernatant samples of the different strains. Immunodetection was performed with specific polyclonal anti-YopH, -YopD and -YopE antibodies.

Next, to establish the effect of OmpR deficiency on the abundance of Yops secreted into the medium, we analysed their release by *Y. enterocolitica* strains Ye9 (wt) and AR4 (*ompR* mutant) following induction by low Ca^2+^ conditions. SDS-PAGE analysis of supernatant samples from a culture of Ye9 grown under inducing conditions (37 °C, BHI-Ca^2+^) confirmed that this strain secretes a series of plasmid-encoded Yop proteins into the growth medium (Figure 4A). As expected, Yops were not produced by strain Ye9 lacking pYV [62], and only trace amounts of some Yops were observed following the growth of Ye9 at 26 °C. The SDS-PAGE Yop profile corresponded well with that described previously [6]. Further examination of the secreted protein profiles revealed that the overall level of Yops was increased in the *ompR* mutant and decreased by complementation of this mutation (AR4/pompR). However, the level of certain proteins secreted by the *ompR* mutant was only slightly increased while others were considerably more abundant compared with the wild-type strain. Interestingly, the band representing YscH/YopR, one of the first proteins to be secreted in T3SS and important in the early stages of needle assembly [15], was absent from the profile of the wild-type strain Ye9 but appeared in that of the *ompR* mutant AR4, confirming that OmpR negatively influences the level of this protein. Western blot analysis of secreted Yops with anti-YopH, -YopD and -YopE specific antibodies showed only a modest increase in their levels in the *ompR* mutant compared with the wild-type strain (Figure 4B). These results demonstrated that OmpR has a negative effect on the secretion of certain Yop proteins.

Together, our SDS-PAGE and Western blot analyses showed that the absence of OmpR causes increases in both the intracellular and secreted Yops in *Y. enterocolitica*, indicating that this regulator negatively influences the expression/secretion of Ysc-Yop T3SS.

### 2.4. Alteration of Ysc-Yop T3SS Gene Transcription by OmpR

**Figure 5:**
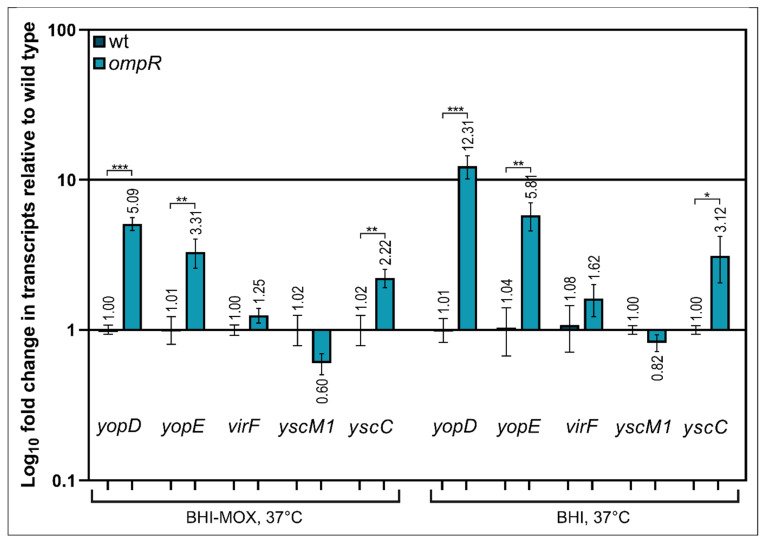
Effect of the *ompR* mutation on the expression of Ysc-Yop T3SS genes. Levels of the mRNAs encoding T3SS-related proteins in the wild-type strain Ye9 and the *ompR* mutant strain AR4 were evaluated by RT-qPCR: regulators VirF and YscM1, effector protein YopE, translocator protein YopD and secretin YscC. Relative *yopD*, *yopE*, *virF*, *yscM1* and *yscC* transcript levels were normalized to the amount of 16S rRNA, taking the mRNA level in Ye9 as 1. Values presented are the mean ± standard deviation from three independent experiments. Significance was determined using Student’s *t*-test (* indicates significance at *p* < 0.05; ** at *p* < 0.01; *** at *p* < 0.001).

It was previously shown that the VirF protein of *Y. enterocolitica* (LcrF in *Y. pseudotuberculosis*) is a master transcriptional activator that directly controls the expression of several T3SS genes/operons by binding to their promoter regions, e.g., *yopE, yopH, virC* and the operons *lcrGVsycD-yopBD* [29]. We hypothesized that OmpR may directly or indirectly affect the expression of the transcriptional activator VirF. To test this notion, we measured *virF* transcript levels by RT-qPCR in the wild-type strain Ye9 and *ompR* mutant AR4, grown at 37 °C in the presence (BHI) or absence of Ca^2+^ (BHI-MOX) (Figure 5). However, levels of the *virF* transcript were not significantly different in the two strains grown in either medium. Next, we investigated the transcriptional response of some *ysc-yop* genes, i.e., *yscC, yopD, yopE* and *yscM1,* in the wild-type and *ompR* strains (Figure 5). The *yscC* gene encodes the translocon component of the T3SS (the OM ring of the basal body of the injectosome), *yopD* and *yopE* encode secreted substrates of T3SS (translocator and effector proteins, respectively), and *yscM1* (*lcrQ* in *Y. pseudotuberculosis*) encodes a protein involved in regulating the expression and function of the T3SS (the anti-activator of *virF* expression in the presence of Ca^2+^) [73]. RT-qPCR data showed that the *yopD* mRNA was expressed at a higher level in the *ompR* mutant than in the wild-type, in both the presence (~12.0-fold) and absence of Ca^2+^ (~5.0-fold). Similarly, levels of the *yopE* transcript were increased in the *ompR* mutant, by ~5.8-fold (+Ca^2+^) and ~3.3-fold (−Ca^2+^), as were the levels of the y*scC* transcript, by ~3-fold (+Ca^2+^) and ~2-fold, (−Ca^2+^). Of the analysed genes, only *yscM1* showed no clear change in its transcript level under both applied conditions.

Together, these results showed that the effect of OmpR on the transcription of Ysc-Yop T3SS genes differs, with the order of influence being *yopD* > *yopE* > *yscC*. This indicated that OmpR-mediated regulation of their expression is complex and does not rely on a single mechanism. We were unable to detect significant changes in the transcript levels of two regulatory genes, *virF* and *yscM1*, which indicated that their expression is not affected by OmpR.

### 2.5. OmpR Binds to the lcrGVsycD-yopBD Operon Promoter Region

**Figure 6:**
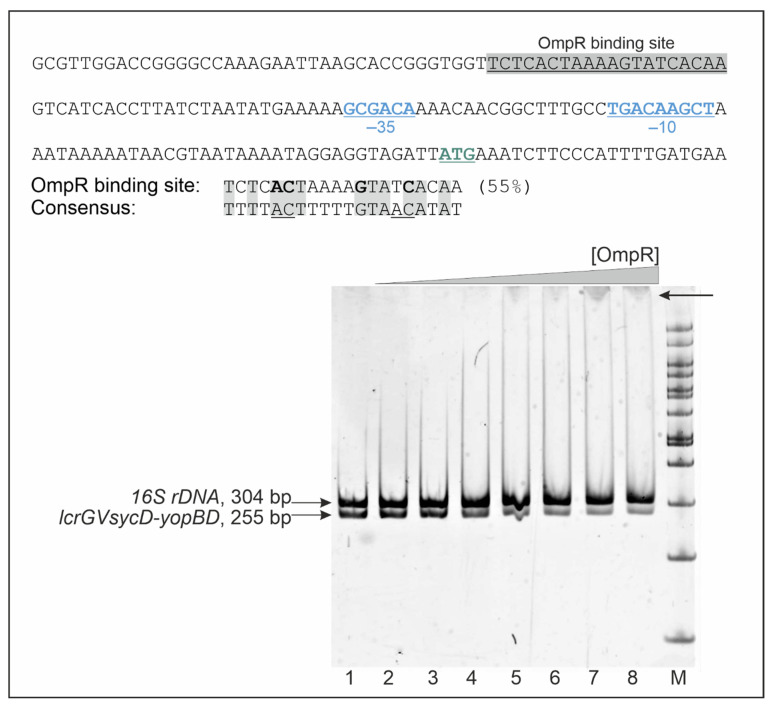
Binding of OmpR to the *lcrGVsycD-yopBD* operon promoter carrying a putative OmpR DNA-binding motif. The sequence of the promoter region of the *lcrGVsycD-yopBD* operon is presented in the upper panel with the −35 and −10 core promoter elements (blue, underlined) and the start codon (ATG, green underlined) indicated. The sequence shaded gray corresponds to the putative OmpR-binding site identified by in silico analysis. The percentage identity to the *E. coli* consensus OmpR-binding sequence is given. The lower panel shows an EMSA confirming the specific binding of OmpR-His_6_ to a 255-bp DNA fragment containing the *lcrGVsycD-yopBD* promoter region. A 304-bp 16S rDNA fragment was used as a control for binding specificity and a competitor DNA. These DNA fragments were incubated with increasing amounts of His_6_-OmpR: 0.6 (lane 2), 1.2 (lane 3), 2.4 (lane 4), 4.8 (lane 5), 6.0 (lane 6), 7.8 (lane 7), 8.0 μM (lane 8) or without protein (lane 1). Protein-DNA complexes were separated by electrophoresis on a 5% native polyacrylamide gel and detected by SYBR Green staining. An arrow indicates a shifted band containing the *lcrGVsycD-yopBD* promoter fragment. M-Nova DNA ladder 100 bp (Novazym). The DNA fragments were amplified with primers listed in Supplementary Table S1.

To gain an insight into the mechanism by which OmpR negatively regulates the expression of *yopD*, *yopE* and *yscC*, we first examined the promoter regions of these genes to identify potential binding sequences for OmpR. Previous analyses of DNA-binding sites of *E. coli* OmpR have defined a 20-bp consensus sequence with the central motif GXXAC or GXXXC, and AC or C nucleotides located about 10 nt away from the AC element of this motif [74,75]. The Ysc-Yop T3SS genes are located in several loci of plasmid pYV and are organized in single transcriptional units or operons. The YopD protein is encoded within operon *lcrGVsycD-yopBD*, YscC within operon *virC* (*yscA-M*), and YopE is encoded by a single gene [25,29,76]. In silico analysis identified a putative 20-bp OmpR-binding sequence upstream of the -35 core promoter of the *lcrGVsycD-yopBD* operon (−27 nt) (Figure 6), indicating that OmpR may influence *yopD* expression directly. However, neither the promoter region of the *yopE* gene nor that of the *virC* operon (*yscC* within the *yscA-M* cluster) contains putative OmpR DNA-binding motifs, so OmpR most likely exerts its regulatory effect in an indirect manner. In addition, we were unable to identify potential OmpR-binding sites within the promoter regions of *yscM1* or the *yscW-virF* operon. 

To investigate whether OmpR directly regulates the expression of YopD by binding to the putative OmpR-binding site identified in the promoter region of the *lcrGVsycD-yopBD* operon, we performed electrophoretic mobility shift assays (EMSA) (Figure 6). Different amounts of purified OmpR-His_6_ were incubated with a 255-bp DNA fragment corresponding to the *lcrGVsycD-yopBD* promoter region plus a 304-bp 16S rDNA fragment as a nonspecific competitor. Specific OmpR binding caused a detectable shift in the migration of the *lcrGVsycD-yopBD* promoter fragment but not of the nonspecific competitor DNA. This result suggested that OmpR binding to the promoter of the *lcrGVsycD-yopBD* operon may regulate its activity and thereby influence YopD expression.

### 2.6. The Expression/Secretion of Yop Proteins in the ompR Mutant Is Correlated with the Amount of Regulatory Protein FlhDC

While the role of OmpR in the expression of Ysc-Yop T3SS genes remained unclear, our results suggested its direct influence on *yopD* and indirect influence on genes encoding other proteins associated with this secretion system. It was previously demonstrated that there is a link between the *Y. enterocolitica ysc-yop* T3SS and the *flhDC* operon encoding the master activator of flagellar genes [77]. The regulator FlhDC resides at the top of the cascade of flagellar genes that are expressed in a stepwise manner to allow proper flagellum assembly [78]. In addition, our previous study showed that OmpR positively regulates the expression of the *flhDC* operon in *Y. enterocolitica* strain Ye9 [61]. Thus, we hypothesized that there might be a link between OmpR-dependent Ysc-Yops production and FlhDC. To identify this potential regulatory circuit, we compared Yops secretion/expression in the wild-type strain Ye9 (wt) with that in mutant strains AR4 (*ompR* mutant) and DN1 (*flhDC* mutant). The *flhDC* mutant was constructed previously by insertional mutagenesis. In this strain, the suicide vector pDS132 containing a 300-bp fragment of *flhDC* was inserted into the genome of strain Ye9 by homologous recombination [62]. Four additional strains were included in this analysis: AR4 complemented with plasmid pHR4 carrying the *ompR* gene (strain ARR12), [59]; DN1 complemented with plasmid pBF expressing the *flhDC* operon from the exogenous p*_lac_* promoter of plasmid pBBR1MCS-3 [62,79]; AR4 carrying the plasmid pBF (*ompR*/pBF) and AR4 carrying empty vector pBBR1MCS-3 (*ompR*/pBBR1).

**Figure 7:**
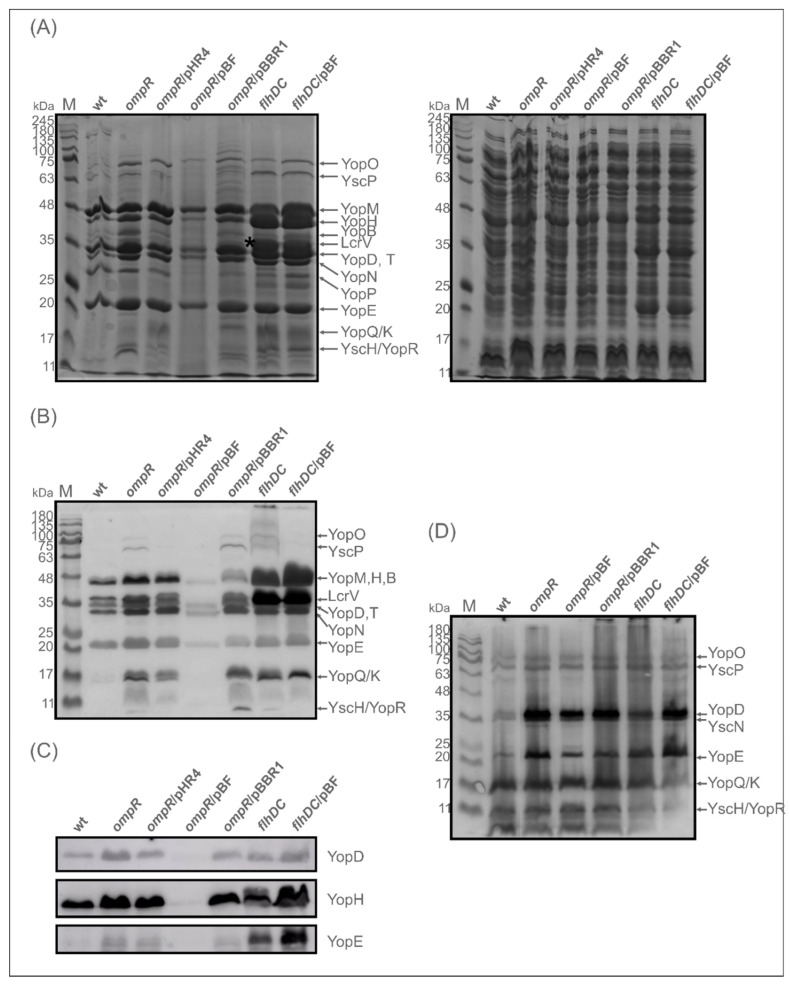
Expression and secretion of Yops by *Y. enterocolitica ompR* and *flhDC* mutants, and by these mutant strains overexpressing *flhDC*, were examined using 12% SDS-PAGE and Western blotting. (**A**) Coomassie blue-stained gel of secreted Yops (left panel) and cell extracts (right panel) of the following strains: wt (Ye9N), *ompR* (AR4), *ompR*/pHR4 (ARR12; mutant AR4 complemented with *ompR*), *ompR*/pBF (AR4/pBBR1MCS-3 carrying *flhDC*), *ompR*/pBBR1 (AR4 with empty vector pBBR1MCS-3), *flhDC* (DN1), *flhDC*/pBF (mutant DN1 complemented with *flhDC*). Proteins from equal numbers of cells were examined. An asterisk indicates protein LcrV was identified in the *flhDC* mutant by LC-MS/MS. (**B**) Immunodetection of secreted Yops with an anti-Yops antiserum. (**C**) Immunodetection of secreted Yop proteins with anti-YopD, -YopH and -YopE antibodies. M-molecular weight standard (Perfect Tricolor Protein Ladder, EURx). Yops were induced by incubation of bacteria for 4 h at 37 °C in Ca^2+^-deprived medium (BHI-MOX). (**D**) Immunodetection of Yops in cell extracts with anti-Yops antiserum. Cell extracts were derived from the strains grown at 37 °C in a standard BHI medium.

As shown in Figure 7A, strain AR4, lacking OmpR, secreted higher amounts of Yops into the culture medium deprived of calcium than the wild-type strain Ye9. Similar to the *ompR* mutant, the *flhDC* mutant (strain DN1) secreted higher amounts of Yops than Ye9. Moreover, DN1 displayed an even greater abundance of most Yops than AR4. Notably, the levels of some Yop proteins, e.g., YopE, were the same in strains DN1 and AR4. In addition, there was a thick band of protein at 35 kDa in the Yops profile of DN1 (marked with an asterisk in Figure 7A), which was not present in that of AR4. This upregulated protein was identified by LC-MS/MS analysis as LcrV. Interestingly, the expression of *flhDC* from pBF (pBBR1-*flhDC*) in the OmpR-deficient strain AR4 caused a decrease in Yops secretion to below the wild-type level, while the vector alone (pBBR1MCS-3) had no such effect on the abundance of Yops produced by this mutant. Complementation of the *ompR* mutation with plasmid pHR4, carrying the gene coding for OmpR (strain ARR12), decreased the Yops level, but unexpectedly the phenotype of the *flhDC* mutant was not changed by introducing a copy of *flhDC* on plasmid pBF. 

The secretion of Yops was further analysed by Western blotting with anti-Yops antiserum (Figure 7B), and specific anti-YopD, -YopH and -YopE antibodies (Figure 7C). This analysis showed that the *flhDC* mutant secretes more Yop proteins than the wild-type strain and the *ompR* mutant. However, of the three Yop proteins examined individually, the level of YopD was not visibly changed. To determine whether FlhDC influences the expression of Yops, total cell extracts were analysed. It is noteworthy that while the *ompR* mutation increased Yops expression, the *flhDC* mutation did not have this effect (Figure 7D). However, the expression of *flhDC* from plasmid pBF in mutant DN1 did upregulate Yops expression. Based on the effect on Yops expression and secretion caused by *flhDC* expressed in multicopy, it may be speculated that the role FlhDC performs in *Y. enterocolitica* is more global. Together, these findings suggested that the secretion of Yops is increased in OmpR-deficient strain AR4, partly as a consequence of decreased levels of FlhDC.

### 2.7. The Relevance of FlhDC Expressed in Trans in the OmpR- and FlhDC-Deficient Strains on Y. enterocolitica Motility

**Figure 8:**
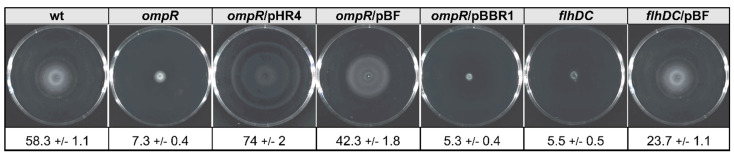
OmpR and FlhDC regulators are required for *Y. enterocolitica* swimming motility. Swimming zones were observed on 0.3% TB0 agar plates after 18 h incubation at 25 °C. Average swimming halo diameters (mm) and standard deviations are presented.

FlhDC is the master activator of the flagellar regulon genes and *Y. enterocolitica* strains lacking this factor exhibit a non-motile phenotype [77]. Greatly reduced motility is also a feature of a *Y. enterocolitica* Ye9 mutant strain lacking OmpR [61]. To examine whether FlhDC expressed *in trans* could influence the motility phenotypes of OmpR- and FlhDC-deficient strains, we performed swimming motility assays with the panel of *Y. enterocolitica* strains used for the analysis of Yops expression/secretion (Figure 8). A motility assay was performed by growing these strains on semi-solid TB0 agar plates at 25 °C. As expected, the motility of the *ompR* mutant was almost completely arrested due to the reduction in *flhDC* expression. Complementation of the *ompR* mutant AR4 with plasmid pHR4 (strain ARR12) restored the swimming motility and increased it above that of the wild-type strain Ye9N. This effect was not observed in strain AR4 harboring empty vector pBBRMCS-3. A clear difference in motility was seen between strain AR4/pBF, expressing wild-type *flhDC* in multicopy, compared with the parent AR4. The introduction of pBF increased the motility of AR4 to the level of the wild-type. The *flhDC* mutant strain DN1 was non-motile, but complementing the mutation with plasmid pBF restored this motility. This result confirmed that *flhDC* expressed from the plasmid pBF is active (even though it did not influence the increased Yops secretion phenotype of this mutant described above) and can complement the non-motile phenotype caused by the *ompR* and *flhDC* mutations.

## 3. Discussion

Experimental studies to define the OmpR regulon of enterobacteria have typically relied on gene expression or microarray and ChIP-chip analysis [52,53]. Previously, we applied a proteomic approach to define the “final” results of OmpR-dependent regulation of OM proteins in *Y. enterocolitica* [65]. The OM proteomes of *Y. enterocolitica* strains (wt vs. *ompR*) grown under different temperatures, osmolarity or pH conditions revealed numerous differences in the abundance of integral OM and membrane-associated proteins. The proteomic analysis detected over one hundred OmpR-dependent proteins involved in various biological processes, among them several known to belong to the OmpR regulon. This finding confirmed the ability of the applied methodology to identify alterations in the protein profiles in response to the level of OmpR. This dataset of OmpR-dependent proteins featured several virulence factors, including components of the Ysc-Yops T3SS. This T3SS, encoded on the virulence plasmid pYV, is essential for the ability of human pathogenic *Yersinia* species to cause disease and is conserved among yersiniae [5]. The T3SS proteins detected in our proteomic analysis were upregulated at 37 °C in the wild-type strain, in agreement with previous reports for *Yersinia*. The thermoregulation of Ysc-Yops T3SS gene expression has been investigated previously, and VirF/LcrF was identified as the main activator of direct Ysc-Yop gene transcription at 37 °C [6,25,26].

Our proteomic analysis showed a higher abundance of a dozen Ysc-Yop proteins in the outer membrane fraction of an *ompR* mutant compared with that of the wild-type parent strain, when grown at 37 °C in different conditions of osmolarity and pH. This suggested that the inhibitory effect of OmpR might play a role in the adaptation of *Y. enterocolitica* to different environmental niches. The T3SS proteins showing increased levels in the *ompR* mutant included the integral OM protein YscC, the major component of the basal body of the Ysc secretory apparatus, and several proteins involved in biogenesis and function of the injectosome (YscP, YscX, YopN, YscN, YopQ). It has been suggested that the formation of the injectosome starts with the incorporation of secretin YscC into the outer membrane [68]. Interestingly, the OmpR-dependent proteins of the Ysc-Yop T3SS included two functional classes of secreted substrates: the translocators YopB and YopD, which facilitate the translocation of proteins across the host cell plasma membrane, and all six Yop effectors known to be delivered into target cells [5]. The abundance of all identified Yops was increased by the deletion of OmpR. The identification of both translocators and the entire set of effector proteins in the outer membrane fraction was unexpected, although localization of Yop effectors of *Yersinia* T3SS as well as Ipa effectors of *Shigella* T3SS in this fraction has been reported previously [23,80]. In addition, more recent studies suggest that the translocators and effectors might be associated with the bacterial cell surface, and their release and polarized translocation are triggered in vivo by contact of the bacterium with the host cell [16]. This proposed two-step model for the delivery of Yops into host cells is an alternative to a one-step injection model in which substrates are directly translocated from the bacterial cytoplasm into the target cell [17]. 

The growth conditions employed for the proteomic analysis were not designed to optimize Yops expression and secretion because the culture medium was not depleted of calcium. Subsequently, we examined the impact of OmpR on the abundance of Yop proteins in samples of OM, total extracts and culture supernatants of cells grown in the absence of calcium ions. These studies showed that overall, Yops secretion/expression was increased in the *ompR* mutant background, particularly in the case of the YopD protein. Our findings indicated that OmpR had a global effect on the Ysc-Yop proteins and, additionally, on the individual components of the T3SS. The impact of OmpR on the abundance of Ysc-Yops proteins might result from its influence on the expression of putative regulators of *ysc-yop* expression/secretion. Two main circuits regulating the expression of ysc-*yop* genes have been proposed. The positive regulation that occurs at 37 °C involves VirF, an activator of *ysc* and *yop* genes, while the negative regulation of these genes results from an accumulation of regulatory protein YscM1 (LcrQ of *Y. pseudotuberculosis*) [5,73]. However, we were unable to detect any differences in the transcript levels of *virF* or *yscM1* resulting from OmpR deficiency, which suggested that other regulatory mechanisms or factors affected by OmpR are responsible for the observed alterations in Ysc-Yop proteins. Interestingly, the strong negative influence of OmpR on the *yopD* transcript level correlated with the effect observed at the YopD protein level. In addition, we were able to identify a potential OmpR-binding site in the promoter region of the *lcrGVsycD-yopBD* operon, which suggests a direct role for OmpR in regulating *yopD* expression. The highly repressive effect of OmpR on YopD expression might influence the level of Yops expression/secretion. The multiple functions of YopD include the ability to inhibit or enhance the expression of LcrF, the main activator of *ysc-yops* genes. Previous studies have implicated YopD together with its chaperone LcrH in the repression of T3SS gene expression [20,21,81]. The inhibition of T3SS gene expression under non-inducing conditions appears to result from the role of the YopD-LcrH complex in the post-transcriptional repression of *lcrF* [81,82]. In turn, the positive effect of YopD on Yops production might depend on its impact on the translational regulator CsrA [83]. Thus, OmpR might influence the regulatory function of YopD to optimize precise usage of the Ysc-Yops T3SS in different environmental niches during the process of infection. Interestingly, two other TCSs in *Yersinia*, namely CpxA/CpxR and the Rcs phosphorelay, modulate the Ysc-Yop T3SS by inversely regulating *lcrF* expression [32,35,36]. 

To further characterize the mechanism underlying the global *ysc-yops* upregulation in the *ompR* mutant AR4, we focused on the role of FlhDC in the OmpR-dependent regulation of T3SS. Upregulation of the *yop* regulon in *Y. enterocolitica* bioserotype 2/O:9 by deletion of *flhDC*, encoding the master activator of the flagellar secretion apparatus, was observed previously [77]. In addition, positive regulation of *flhDC* expression by OmpR has been demonstrated in *Y. enterocolitica* strain Ye9 [61] and in *Y. pseudotuberculosis* [55]. Therefore, we hypothesized that the downregulation of *flhDC* in the *ompR* mutant could cause the increased production of Ysc-Yops. Focusing on the role of FlhDC in the OmpR-dependent regulation of T3SS, we confirmed the upregulation of Yops secretion in an *flhDC* mutant of *Y. enterocolitica* Ye9, reported previously in strain MRS40 [77]. In addition, the elevated level of Yops in the OmpR-deficient strain was suppressed by FlhDC expressed *in trans*. The regulatory circuit involving both OmpR and FlhDC was also demonstrated with a motility assay. The overexpression of *flhDC* in a non-motile *ompR* mutant of *Y. enterocolitica* led to hypermotility of this strain. The crosstalk between the *ysc-yop* genes and *flhDC* in *Y. enterocolitica* was explained by Bleves and co-workers [77] through the role of FlhDC in the downregulation of *virF* expression. If such regulation occurs, there should be some influence of OmpR on *virF* expression, but we did not detect this. It is possible that some other unknown mechanism may mask such regulation. Interestingly, the regulatory link between FlhDC and the *ysc-yop* regulon was not observed in a *Y. enterocolitica* strain of bioserotype 1B/O:8 [84]. However, this is not surprising because strains of bioserotype 1B/O:8 differ from those of bioserotype 2/O:9 in many physiological aspects [66,85].

In the present study, we have shown that the upregulation of *ysc-yop* T3SS in the *ompR* mutant correlates with a decrease in the FlhDC directed transcription of genes necessary for the structure and assembly of the flagellar secretion apparatus (T3SS) [86]. The virulence- and flagellar-associated T3SSs have analogous structures, with many proteins that exhibit similarity in sequence or function [10,87,88]. The loss of OmpR might result in a decrease in the flagella secretion machinery in the OM, which could change its structure, thereby leading to a higher level of Ysc secretion apparatus and therefore increased Yops secretion. Thus, by interfering with the assembly and function of the flagellar T3SS, OmpR might indirectly affect the production and function of the Ysc-Yop T3SS. 

## 4. Materials and Methods

### 4.1. Bacterial Growth Conditions

Unless indicated, *Y. enterocolitica* strain Ye9 and its derivatives were routinely grown at 26 °C in lysogeny broth (LB, BioShop, Burlington, ON, Canada) or on LB agar. To promote the secretion of plasmid pYV-encoded proteins (Yops), *Y. enterocolitica* strains were cultivated at 37 °C in brain-heart infusion (BHI, Merck Millipore, Burlington, MA, USA) medium with 20 mM sodium oxalate and 20 mM MgCl_2_ to cause the depletion of Ca^2+^ ions (BHI-MOX). Bacteria carrying antibiotic resistance markers were grown in the presence of the appropriate antibiotics: chloramphenicol (Cm)—25 μg mL^−1^, gentamicin (Gm)—40 µg mL^−1^, kanamycin (Km)—50 μg mL^−1^, nalidixic acid (Nal)—30 μg mL^−1^, tetracycline (Tet)—12.5 μg mL^−1^. For proteomic experiments, triplicate overnight cultures of wild-type strain Ye9 and its *ompR* mutant derivative AR4 were grown in LB at pH 7.0 with 86 mM NaCl (standard medium), LB adjusted to pH 5.0 by the addition of 100 mM HOMOPIPES buffer [homopiperazine-N,N′-bis-2-(ethanesulfonic acid)] or in LB at pH 7.0, supplemented with NaCl to 386 mM.

### 4.2. Isolation of Outer Membrane-Enriched Sarkosyl-Insoluble Fractions and Proteomic Analysis

A detailed description of the isolation of outer membrane-enriched sarkosyl-insoluble fractions of *Y. enterocolitica* cells for shotgun label-free quantitative proteomic analysis has been presented previously [65]. Briefly, triplicate cultures of strains Ye9 and AR4 were incubated at 26 °C or 37 °C in standard LB medium (86 mM NaCl, pH 7.0); LB adjusted to pH 5.0 or supplemented with NaCl (386 mM, pH 7.0) were centrifuged and the cell pellets were used for fractionation. Following sonication and ultracentrifugation of the samples, the bacterial membrane extracts were treated with sarkosyl, which preferentially solubilizes proteins associated with the inner membrane. Sarkosyl-insoluble protein fractions (OMsl) were subsequently solubilized using a combination of the detergents SDS (0.1%) and sodium deoxycholate (1%). The extracts were then subjected to digestion with trypsin and analysed qualitatively and quantitatively by mass spectrometry. Shotgun label-free quantitative LC-MS/MS analysis of all samples was used to produce a dataset of membrane proteins or proteins functionally connected with the OM fraction. The proteomes obtained from strains grown under different conditions were compared to generate differential OMsl proteome lists [65].

### 4.3. Induction and Analysis of Secreted Yop Proteins

The induction and analysis of secreted Yop proteins were performed, essentially as described previously [89]. Strains were grown overnight in a BHI medium at 26 °C. These cultures were diluted in BHI-MOX to an optical density of 600 nm (OD_600_) of 0.1 and incubated further at 26 °C. Once an OD_600_ of 0.3 was attained, the cultures were transferred to 37 °C and incubated with shaking (150 rpm) for 4 h. After measuring the OD_600_, the bacteria in each culture were harvested by centrifugation at 4000× *g* for 15 min at 4 °C. The supernatants were filtered (0.22 μm filter, Merck Millipore, Burlington, MA, USA), trichloroacetic acid was added to a final concentration of 5% and the proteins were precipitated by holding overnight at 4 °C. The pellets formed by centrifugation at 13,000× *g* for 20 min at 4 °C were washed with ice-cold 80% acetone. The Yop protein preparations were dissolved in a volume of 2× Laemmli sample buffer determined by the OD_600_ value of the culture and analysed by electrophoresis on 12% SDS-polyacrylamide gels. 

### 4.4. Western Blotting

For the abundance of Yop proteins in OM protein fractions prepared for shotgun proteomic analysis [65], total bacterial protein extracts and cell culture supernatants were evaluated by Western blotting. Equivalent samples, according to the OD_600_ reading, were taken from each culture for analysis by SDS-PAGE. Next, the proteins were transferred to nitrocellulose membranes (Amersham Protran Western blotting membrane, GE Healthcare, Chicago, IL, USA) using a wet electroblotting system (Bio-Rad, Hercules, CA, USA). The blots were then probed with a rabbit antiserum directed against multiple Yops (diluted 1:1500) (Faculty of Biology, University of Warsaw, [89]) or specific anti-YopD, -YopH and -YopE polyclonal antibodies (diluted 1:2000, 1:2000 and 1:5000, respectively), obtained from the Max von Pettenkofer Institute for Hygiene and Medical Microbiology (University of Munich). As the secondary antibodies, goat anti-rabbit IgG, conjugated to alkaline phosphatase (Sigma-Aldrich, Saint Louis, MO, USA) or sheep anti-rabbit IgG, conjugated to peroxidase (Sigma-Aldrich, Saint Louis, MO, USA) were used (diluted 1:30,000 or 1:15,000, respectively). The positive immunoreaction was visualized using the chromogenic substrate 5-bromo-4-chloro-3-indolyl phosphate/nitro blue tetrazolium chloride (BCIP/NBT; Sigma-Aldrich, Saint Louis, MO, USA) or Clarity Western ECL Substrate (Bio-Rad, Hercules, CA, USA). The loading of equivalent amounts of proteins was controlled by Coomassie blue staining of an identical gel. In addition, Ponceau S staining was used to disclose the protein bands on Western blot membranes.

### 4.5. RT-qPCR

Total RNA was isolated from cultures of *Y*. *enterocolitica* strains cultivated in BHI medium at 37 °C, as well as from cells after the induction of Yops secretion in calcium-deprived medium BHI-MOX (each culture in triplicate). After cultures reached OD_600_ ~1, the cells were harvested by centrifugation and RNA was isolated from the cell pellets using a Nucleo Spin RNA purification kit (Macherey-Nagel, Düren, Germany). Isolated RNAs were DNase treated with a TURBO DNA-free Kit (Invitrogen, Carlsbad, CA, USA). The purity and quality of the RNA were assessed using a Qubit 4 with the Qubit RNA IQ Assay Kit (Thermo Fisher Scientific, Waltham, MA, USA). An amount of 1 µg of each RNA was used as a template for cDNA synthesis with a Maxima H Minus First Strand cDNA Synthesis Kit (Thermo Fisher Scientific, Waltham, MA, USA). A quantity of cDNA corresponding to 10 ng of input RNA was used as the template in each RT-qPCR. Oligonucleotide primers used for qPCR were purchased from Genomed S.A. (Warsaw, Poland) and are listed in Table S1. The qPCR was conducted using a LightCycler 480 II (Roche, Basel, Switzerland) with 5× HOT FIREPol EvaGreen qPCR Mix Plus (Solis Biodyne, Tartu, Estonia). The levels of the amplified PCR products were normalized to that of a fragment amplified from the 16S rRNA reference gene. Fold changes were calculated using the Pfaffl method [90].

### 4.6. Electrophoretic Mobility Shift Assay (EMSA)

Overproduction and purification of OmpR-His_6_ and its use in an EMSA to detect interactions of this recombinant protein with DNA sequences were performed as described previously [64]. Briefly, a 255-bp fragment of the promoter region of the *lcrGVsycD-yopBD* operon and a 304-bp 16S rDNA fragment as a nonspecific competitor were PCR-amplified using *Y. enterocolitica* genomic DNA as a template and the pairs of primers listed in Table S1. The DNA fragments were incubated in OmpR-binding buffer (50 mM Tris-HCl pH 8.0, 100 mM KCl, 1 mM EDTA, 1 mM DTT, 20 mM MgCl_2_, 12% glycerol, 100 µg/mL BSA, 0.1% Triton X-100) with increasing amounts of OmpR-His_6_ (molar ratio of 1:20–300) at 26 °C for 30 min. The samples were then loaded on a 4.2% native polyacrylamide gel (19:1 acrylamide/bisacrylamide, 0.2 × TBE) containing 2% glycerol and electrophoresis performed at 110 V for approximately 3 h. The gel was then stained in 1 × SYBRgreen solution (Invitrogen, Waltham, MA, USA) and visualized using a GE Healthcare AI600 imager (GE Healthcare, Chicago, IL, USA).

### 4.7. Motility Assay

*Y. enterocolitica* strains were cultured overnight in LB at 26 °C. Aliquots (4 μL) of each culture were spotted onto soft agar TB0 motility plates (TB medium without NaCl containing 1.0% tryptone and 0.3% agar) and incubated at 25 °C. After 18 h, the motility zones were measured and photographs were taken using a GE Healthcare AI600 imager. 

### 4.8. Tandem Mass Spectrometry (MS/MS) Analysis

Selected protein bands observed on stained SDS-polyacrylamide gels were excised, digested with trypsin and subjected to liquid chromatography-tandem mass spectrometry (LC-MS/MS), (IBB PAN, Warsaw, Poland).

### 4.9. Bioinformatic Data and Statistical Analysis

LC-MS data were qualitatively and quantitatively analysed. All software used is described in detail by Nieckarz et al. [65]. The names of the Ysc-Yop T3SS genes (and their encoded proteins) in the genome of *Y. enterocolitica* Ye9 bioserotype 2/O9 were assigned based on the names of orthologous genes/proteins in *Y. enterocolitica* subsp. *palearctica* 105.5R(r), *Y. enterocolitica* subsp*. enterocolitica* 8081 and related *Yersinia* species (*Y. pseudotuberculosis* and *Y. pestis*). Complete genome sequences of *Yersinia* were examined in GenBank (http://www.ncbi.nlm.nih.gov (accessed on 1 September 2015). Gene ontology data were obtained from the UniProt databases (http://www.uniprot.org (accessed on 1 September 2015). In silico analyses of putative OmpR-binding sites in the promoter regions of *ysc-yops* genes were performed using a shotgun genome sequence of *Y. enterocolitica* subsp. *palearctica* Ye9N (bioserotype 2/O:9; NCBI/GenBank: JAALCX000000000).

Statistical analyses were performed using Prism 7 software (v.7.02, GraphPad, San Diego, CA, USA). For RT-qPCR experiments, significance was calculated using the Student’s unpaired *t*-test. A *p*-value of <0.05 was considered significant. All experiments were performed two or more times unless stated otherwise.

## 5. Conclusions

Human pathogenic *Yersinia* species carry plasmid pYV, encoding the Ysc-Yop type III secretion system (T3SS), which is important for the virulence of these bacteria. This study revealed that OmpR, the regulator in the EnvZ/OmpR two-component system, negatively controls the expression and secretion of Ysc-Yop proteins in *Y. enterocolitica*. Downregulation of the Ysc-Yop type III secretion system (T3SS) by OmpR involves changes in the level of translocatory/regulatory protein YopD and FlhDC, the master regulator of flagellar-associated T3SS expression. The positive effect of OmpR on *flhDC* expression results in the inverse regulation of motility and Yops expression/secretion. These results suggest that flagellar T3SS assembly antagonizes the virulence T3SS and that a negative crosstalk exists between these two systems, probably to maintain the stability and functional features of the cell envelope. Thus, OmpR might guard the integrity of the bacterial cell envelope by regulating two separate secretion systems. Such control may play a role in the precise expression of the appropriate T3SSs according to the environmental conditions and thus, contribute to the fitness and virulence of *Y. enterocolitica*.

## Figures and Tables

**Table 1:** Ysc-Yop T3SS proteins produced by *Y*. *enterocolitica* wild-type strain Ye9 and isogenic *ompR* mutant AR4 grown at 37 °C under different physicochemical conditions.

Differentially Expressed Proteins		Regulation Ye9 vs. AR4 ^b^											
		Standard Conditions				High Osmolarity				Low pH			
Accession Number (GenBank)	Protein Description ^a^	*q*-Value ^c^	Ratio ^d^	fc ^e^	pep ^f^	*q*-Value	Ratio	fc	pep	*q*-Value	Ratio	fc	pep
ADZ44444	Translocator protein YopB	0.00005	0.02	48.12	41	0.00007	0.02	42.4	42	0.00005	0.01	75.73	41
ADZ44443	Translocator protein YopD	0.00005	0.02	40.37	48	0.00007	0.03	37.76	48	0.00005	0.01	71.6	48
ADZ44440	Effector protein YopM	0.00073	0.05	20.79	23	0.00276	0.02	42.45	21	0.07599	0.25	3.95	22
ADZ44434	Negative modulator of injection YopQ/YopK	0.00005	0.05	18.54	18	0.0003	0.05	21.11	17	0.00005	0.03	33.89	18
ADZ44516	Protein kinase YopO	0.00005	0.06	16.25	64	0.00007	0.1	9.64	57	0.00005	0.04	22.77	61
ADZ44454	OM protein YopN	0.00147	0.07	14.62	12	0.04588	0.07	14.4	10	0.01486	0.06	17.46	11
ADZ44451	Protein YscX	0.0194	0.08	11.89	6	0.34986	0.18	5.52	6	0.13906	0.17	5.83	6
ADZ44518	Effector protein YopP	0.00048	0.1	10.37	26	0.00057	0.08	11.94	25	0.0019	0.05	19.12	25
ADZ44467	OM pore forming protein YscC	0.00005	0.13	7.48	31	0.00007	0.16	6.37	31	0.00005	0.13	7.66	31
ADZ44479	Tyrosine-protein phosphatase effector protein YopH	0.00005	0.15	6.78	76	0.00007	0.12	8.49	69	0.00005	0.08	13.32	72
EOR65641	Effector protein YopE	0.00005	0.17	5.77	29	0.00007	0.12	8.41	27	0.00005	0.07	15.03	30
ADZ44435	Effector protein YopT	0.00998	0.24	4.12	10	0.18349	0.24	4.08	9	0.10699	0.2	4.97	8
ADZ44455	H^+^-transporting two-sector ATPase YscN	0.0128	0.35	2.87	13	0.21592	0.49	2.04	12	0.04486	0.31	3.23	12
ADZ44457	Needle length determinant YscP	0.02605	0.36	2.78	13	0.26892	0.45	2.23	12	0.11434	0.42	2.39	13

Proteins of T3SS are sorted according to the effect of OmpR under standard conditions (ranked from highest to lowest fold change). ^a^ Description of proteins identified in OMsl (outer membrane-enriched sarkosyl-insoluble fractions) according to the UniProt databases, GenBank or homologous sequences obtained using BLAST. Proteins were clustered based on gene ontology (biological process) terms. ^b^ Proteins whose production differed between the wild-type strain Ye9 and OmpR-deficient mutant AR4, grown under different physicochemical conditions, according to shotgun label-free MS analysis. Standard conditions (LB medium); high osmolarity (LB supplemented with NaCl to 386 mM); low pH (LB adjusted to pH 5.0), at 37 °C. ^c^ *q*-value ≤ 0.05—statistically significant differences in production. *q*-value > 0.05 is marked in red. ^d^ Significant changes in protein production are defined by a ratio of abundance of ≤ 0.67 (protein more abundant in *ompR* mutant strain. ^e^ fc—fold change. ^f^ pep—number of identified peptides belonging to the differentially produced protein.

## Supplementary Materials

The following supporting information can be downloaded at: https://www.mdpi.com/article/10.3390/ijms23094758/s1.
