# Supplementary material for: The Regulatory Circuit Underlying Downregulation of a Type III Secretion System in Yersinia enterocolitica by Transcription Factor OmpR
Source: Int J Mol Sci. 2022 Apr 26;23(9):4758. doi: 10.3390/ijms23094758 (PMC9100119; doi:10.3390/ijms23094758)
Supplement: Supplementary file 1 [file ijms-23-04758-s001.zip › ijms-1692951-supplementary.pdf]

Table S1. Oligonucleotide primers used in this study.

Purpose	Gene name	Name of primer	Primer sequence (5′ → 3′)	Reference
RT-qPCR	*yopD*	RTyopDYe9-F	CAAACCGAGTCAGGGAATCA	This study
		RTyopDYe9-R	CCAGTTCCAACAGCAACAAC	
	yopE	RTyopEYe9-F	GGCACCCAGTGTTATGGAAT	This study
		RTyopEYe9-R	TGAGGTTTGTAGCCGTTCAG	
	virF	RTvirFYe9-F	TTTAGGCAACCGCCCAGAAGAA	This study
		RTvirFYe9-R	GAAATGCCATAAACTGTACCAAACA	
	yscM1	RTyscM1Ye9-F	TTCCGATGAGCGCAGATTT	This study
		RTyscM1Ye9-R	AGACGCCCTTGGTAATAGTTTC	
	yscC	RTyscCYe9-F	TTTCACGTCCGACCCTGTTA	This study
		RTyscCYe9-R	TCCCTTTCAGTTCAGCCACT	
	16s rRNA (reference gene)	RT16rRNAYe9-F	CATCATGGCCCTTACGAGTAG	[1]
		RT16rRNAYe9-R	CCGGACTACGACAGACTTTATG	
EMSA, 255-bp fragment of *lcrGVsycD-yopBD* promoter region	*lcrGVsycD-yopBD* promoter region	Lcr-yopFor	CGGGGCCAAAGAATTAAGCA	This study
		Lcr-yopRev	GCCGATATCAGCACACATTTCT	
EMSA, 304-bp fragment of 16S rDNA used as a negative control	16S rDNA	E16S304Ye-F	ATTCCGATTAACGCTTGCAC	[2]
		E16S304Ye-R	GTGGGGTAATGGCTCACCTA	

References

1. Jaworska, K., Ludwiczak, M., Murawska, E., Raczkowska, A., Brzostek, K. The Regulator OmpR in *Yersinia enterocolitica* Participates in Iron Homeostasis by Modulating Fur Level and Affecting the Expression of Genes Involved in Iron Uptake. *Int. J. Mol. Sci*. **2021**, 22(3):1475. doi: 10.3390/ijms22031475.

2. Nieckarz, M., Raczkowska, A., Debski, J., Kistowski, M., Dadlez, M., Heesemann, J., Rossier, O., Brzostek K. Impact of OmpR on the membrane proteome of *Yersinia enterocolitica* in different environments: Repression of major adhesin YadA and heme receptor HemR. *Environ. Microbiol.* **2016**, 18, 997–1021. doi: 10.1111/1462-2920.13165.
